# Spinal disease in myeloma: cohort analysis at a specialist spinal surgery centre indicates benefit of early surgical augmentation or bracing

**DOI:** 10.1186/s12885-016-2495-7

**Published:** 2016-07-11

**Authors:** Karan Malhotra, Joseph S. Butler, Hai Ming Yu, Susanne Selvadurai, Shirley D’Sa, Neil Rabin, Charalampia Kyriakou, Kwee Yong, Sean Molloy

**Affiliations:** Spinal Deformity Unit, Department of Spinal Surgery, Royal National Orthopaedic Hospital, Brockley Hill, Stanmore HA7 4LP UK; Department of Orthopaedics, The Second Affiliated Hospital of Fujian Medical University, Quanzhou City, Fujian Province People’s Republic of China; Department of Clinical Haematology, University College London Hospitals, 235 Euston Road, London, NW1 2BU UK; Department of Clinical Haematology, The Royal Free Hospital, Pond Street, London, NW3 2QG UK

**Keywords:** Multiple myeloma, Vertebral fracture, Outcome scores, Vertebral augmentation, Thoracolumbar bracing

## Abstract

**Background:**

Multiple myeloma osteolytic disease affecting the spine results in vertebral compression fractures. These are painful, result in kyphosis, and impact respiratory function and quality of life. We explore the impact of time to presentation on the efficacy of spinal treatment modalities.

**Methods:**

We retrospectively reviewed 183 patients with spinal myeloma presenting to our service over a 2 year period.

**Results:**

Median time from multiple myeloma diagnosis to presentation at our centre was 195 days. Eighty-four patients (45.9 %) were treated with balloon kyphoplasty and the remainder with a thoracolumbar-sacral orthosis as per our published protocol. Patients presenting earlier than 195 days from diagnosis had significant improvements in patient reported outcome measures: EuroQol 5-Dimensions (*p* < 0.001), Oswestry Disability Index (*p* < 0.001), and Visual Analogue Pain Score (*p* < 0.001) at follow-up, regardless of treatment. Patients presenting after 195 days, however, only experienced benefit following balloon kyphoplasty, with no significant benefit from non-operative management.

**Conclusion:**

Vertebral augmentation and thoracolumbar bracing improve patient reported outcome scores in patients with spinal myeloma. However, delay in treatment negatively impacts clinical outcome, particularly if managed non-operatively. It is important to screen and treat patients with MM and back pain early to prevent deformity and improve quality of life.

**Electronic supplementary material:**

The online version of this article (doi:10.1186/s12885-016-2495-7) contains supplementary material, which is available to authorized users.

## Background

In multiple myeloma (MM), osteolytic disease in the spine is common as the high hematopoietic marrow content of the vertebrae offers an attractive site for localisation and growth of neoplastic plasma cells [1, 2]. Through a variety of signal transduction pathways osteoclasts are preferentially activated and the homeostatic balance of bone remodelling shifts towards resorption [2, 3]. Localised osteoporosis ensues and may result in vertebral body compression fractures (VCFs) [3, 4]. This is potentially exacerbated by high dose steroid treatment used in the treatment of MM, further weakening the bone.

Multiple VCFs and increasing thoracic kyphosis have been shown to adversely affect functional status in the osteoporotic population and are associated with significantly reduced lung function and increased pulmonary complications [5–9]. In the non-osteoporotic adult population, a kyphotic deformity of the spine has also been shown to adversely affect health related quality of life scores [10].

Augmentation of a fractured vertebral body with acrylic cement has been shown to restore its strength and prevent further kyphosis [11–13]. This augmentation can be performed using minimally invasive techniques such as percutaneous vertebroplasty or balloon kyphoplasty (BKP). Both techniques have been shown to significantly reduce pain from VCFs and improve function in patients with metastatic disease and myeloma [14–17]. Functional outcome is particularly important in patients with MM as the life expectancy of this patient cohort continues to increase with the introduction of modern chemotherapeutic treatment regimens [2].

We describe the clinical and radiographic parameters of patients with an established diagnosis of MM presenting to our tertiary referral spinal service, and their response to treatment for their VCFs. We assess response by the change in patient reported outcome scores following intervention. Our objectives are: to explore the way in which spinal deformity affects clinical outcomes, and to explore the impact of time to presentation on the efficacy of spinal treatment modalities.

## Methods

### Patients

This study was performed at a national tertiary centre for the treatment of spinal MM, using a protocol approved by our Institutional Review Board (Research Governance Team, Research & Innovation, Royal National Orthopaedic Hospital, Stanmore, UK; Reference: SE14.019). We routinely collect demographic and clinical outcome data on all patients and patients consent to their data being used for the purposes of research and analysis. Data collected on patients presenting with MM bone disease involving the spinal column between June 2013 and May 2015 was retrospectively analysed. We included all adult patients in whom MM was the primary cause for their VCFs.

Clinical data collected included patient demographics, date of MM diagnosis, number and level of VCFs, treatment given, and time from diagnosis of MM to presentation at our service. We analysed clinical and radiographic outcome variables at time of presentation and at follow-up 6-weeks after treatment. Clinical outcome measures were assessed using patient reported health related quality of life scores as discussed below. Patients were treated either with both BKP and a front-opening thoraco-lumbar-sacral orthosis (TLSO), or with a TLSO alone in line with our published guidelines for management of spinal myeloma (described below) [18].

We excluded patients with missing clinical outcome scores, inadequate radiographs (radiographs not taken according to protocol described below), VCFs due to a diagnosis other than MM, cord compression, or with neurological deficit. We also excluded patients if they had had previous spinal fusion surgery or cement augmentation (vertebroplasty or BKP) prior to presentation at our institution, or if they were lost to follow-up.

### Radiology

All patients were referred with whole spine magnetic resonance (MR) scans. All patients had standardised, full length, standing, lateral spinal radiographs taken at presentation (and 6 weeks post-BKP). Our imaging software took into account and adjusted for magnification when taking measurements on radiographs (calibrated for 5 % magnification). All measurements were done digitally using Patient Archiving and Communication Software (PACS, Sectra, Sweden). Radiographic outcome measures collected included: thoracic kyphosis, lumbar lordosis and sagittal vertical axis. Thoracic kyphosis was measured as the angle between the inferior vertebral body end plate of T12 and the superior end plate of T4. Similarly, lumbar lordosis was measured from the superior endplate of S1 to the superior end plate of L1. Sagittal vertical axis was measured as the horizontal distance from the posterior-superior vertebral body end plate of S1 to a vertical plumb line drawn from the C7 vertebra. Additionally, we recorded kyphosis between T5 to T10 – mid-thoracic kyphosis, and T10 to L2 – thoracolumbar kyphosis. Normal ranges are published and listed in Table 1 [19–22]. Further information on the clinical importance of these measurements and an example case are illustrated in our supplementary data (Additional file 1).

**Table 1:** Results of the radiological parameters recorded at presentation. A negative sagittal vertical axis (SVA) indicates that the centre of gravity of the spine falls behind the superior endplate of S1. The mean thoracic kyphosis (TK) is higher and the mean lumbar lordosis (LL) is lower (more kyphotic) than in the literature reflecting our patient population. It can also be seen that this is mostly due to kyphosis in the mid thoracic (MTK) and thoracolumbar (TLK) regions [19–22]

	TK (°)	LL (°)	SVA (mm)	MTK (°)	TLK (°)
Mean	56.2	48.4	53.5	38.4	21.3
Std. deviation	18.2	16.5	52.1	16.8	16.2
Minimum	9	3	−52	5	−10
Maximum	106	94	198	85	67
Population mean	40 ± 10	56 ± 13	7 ± 32	15 ± 4	1 ± 9

For patients undergoing BKP, vertical height of the vertebral body was measured before and after the procedure and compared to the height of adjacent normal vertebral bodies to obtain the percentage of height lost after VCF, and the percentage of height restored after BKP. These measurements were taken at the anterior border and the mid-point of the vertebral bodies (illustrated in our supplementary data in Additional file 1).

### Interventions

Patients were treated with either TLSO alone, or with BKP and a TLSO. MR scans and clinical examination were used to determine the state of healing of the spinal fractures at time of presentation. Fractures which had completely healed did not require either form of treatment. For those patients with unhealed fractures, the spinal instability neoplastic score (SINS) was used to determine their stability. The SINS score was calculated from the MR scans. For those patients with fractures classed as ‘stable’ (score of 0–6) or ‘impending instability’ (score of 7–12) using the SINS score, a TLSO was prescribed in order to support the vertebral column and prevent further deformity whilst healing occurred. For those patients with fractures classed as ‘unstable’ (score of 13–18) a BKP was performed to prevent further deformity from occurring. Regardless of stability, patients with fractures which were painful were offered BKP, where medically fit for surgery. This was assessed using the visual analogue score, and a score of 6/10 or more was our cut-off. All patients undergoing BKP were also treated with a TLSO post-operatively.

Where a TLSO was used, this was a front-opening orthosis which was worn when the patient was standing or mobilising, but which could be taken off in bed. The brace was worn for a period of 3 months whilst fracture healing occurred. BKP was performed under general anaesthesia, with the patient prone, under fluoroscopic guidance and with antibiotic prophylaxis. Unilateral, para-spinal stab incisions were made and a trochar was introduced into the vertebral body through the pedicle. The balloon was then inflated to create a cavity in the vertebral body and the space then filled with cement. Post-operatively patients were allowed to mobilise in their TLSO and were discharged the following day.

### Outcome measures

Clinical outcomes measures utilised to assess health related quality of life included the validated scoring measures of Euro-Qol 5 Dimensions (EQ-5D), Oswestry Disability Index (ODI), and the Visual Analogue Score for the trunk (VASB). These scores were recorded at the time of initial presentation to our service and were repeated at follow-up 6 weeks after intervention. The minimum clinically important difference in scores was taken as 0.090 points for EQ-5D, 8.8 points for the ODI, and 1.2 points for the VASB [23–25].

### Statistical analysis

Statistical analysis was performed using SPSS 16.0 (IBM, New York, USA). Data are presented as mean ± standard deviation, or as medians with a range. Correlation between radiographic and clinical variables was analysed using Pearson’s coefficient for parametric data and Spearman’s rank correlation for non-parametric data. We also divided patients into groups based on time from diagnosis to presentation (in groups of 30 days intervals) and assessed for differences in radiological and clinical parameters. Comparison between groups was carried out using paired and independent t-tests for parametric data, and Wilcoxon signed ranks and Mann-Whitney U tests for non-parametric. Statistical significance was considered to be a 2-tailed *p*-value <0.05.

## Results

### Patients

One hundred and ninety six patients meeting our inclusion criteria were reviewed in our tertiary spinal MM service between June 2013 and May 2015. Thirteen patients were excluded due to presence of cord compression, neurological deficit, or previous surgery, leaving 183 patients with symptomatic MM of the spine for subsequent analysis.

The median age was 66 years (range: 37–91 years). There was a male preponderance (male:female ratio of 1.69:1). Of the patients seen 136 (74.3 %) had a new diagnosis of myeloma and 47 (25.7 %) presented after a relapse. Ninety-three patients (50.8 %) had IgG subtype MM, 22 (12.0 %) had IgA, 29 (15.9 %) had light chain subtype and 9 (4.9 %) had other subtypes of MM. In 30 patients (16.4 %) the subtype was not known. Sixty five patients (35.5 %) had autologous stem cell transplant prior to presentation. Four patients (2.2 %) were taking oral bisphosphonates at time of presentation and 113 (61.8 %) were being treated with intravenous bisphosphonates. There was no active bisphosphonate treatment recorded for 66 patients (36.0 %) at time of presentation.

The mean number of vertebral fractures secondary to MM at time of presentation was 3.7 ± 2.7 levels. The median time to presentation was 195 days (interquartile range: 72–996 days). The median duration of spinal follow-up was 206 days (interquartile range: 92–418 days). Twenty-two patients (12.02 %) died, at a median time of 258 days from presentation at our unit (range: 97–470 days). The data are summarised in Table 2, along with actual ranges.

**Table 2:** Basic characteristics of patients sampled. Data presented as number of patients in each group or as a median accompanied by full range (interquartile ranges listed in Results section)

Patient characteristic (*N* = 183)	*N* (%) or median (range)
Age	66 years (37–91 years)
Gender:	
Male	115 (62.8 %)
Female	68 (37.2 %)
Disease status:	
Newly diagnosed	136 (74.3 %)
Relapsed	47 (25.7 %)
Chain Isotype:	
IgG	93 (50.8 %)
IgA	22 (12.0 %)
Light chain	29 (15.9 %)
Other	9 (4.9 %)
*Missing*	30 (16.4 %)
Time from diagnosis to presentation	
All patients	195 days (11–5090 days)
New diagnosis of MM	109 days (11–1902 days)
Relapse of MM	1929 days (171–5090 days)
Previous autologous stem cell transplant	65 (35.5 %)
Bisphosphonate therapy	
Intravenous	113 (61.8 %)
Oral	4 (2.2 %)
None recorded	66 (36.0 %)
Acute vertebral compression fractures:	
Number of fractures per patient	3 fractures (0–15 fractures)
Thoracic region (number of patients)	149 (81.4 %)
Lumbar region (number of patients)	111 (60.7 %)
Thoracic and lumbar fractures (number of patients)	85 (46.5 %)
No non-healed fractures	7 (3.8 %)

### Radiological parameters

The mean lumbar lordosis was 48.1° ± 17.2°, mean thoracic kyphosis was 55.2° ± 18.9°, mean sagittal vertical axis was 55.1 mm ± 51.6 mm. The mean mid-thoracic kyphosis was 38.1° ± 17.4°, and thoracolumbar kyphosis was 20.7° ± 15.5°. Table 1 shows the radiological parameters recorded at presentation and the population normal values [19–22]. The mean SINS score at presentation was 12.75 ± 2.03 (range: 6–16). There was no correlation of any radiological parameters with age or sex.

**Fig. 1:**
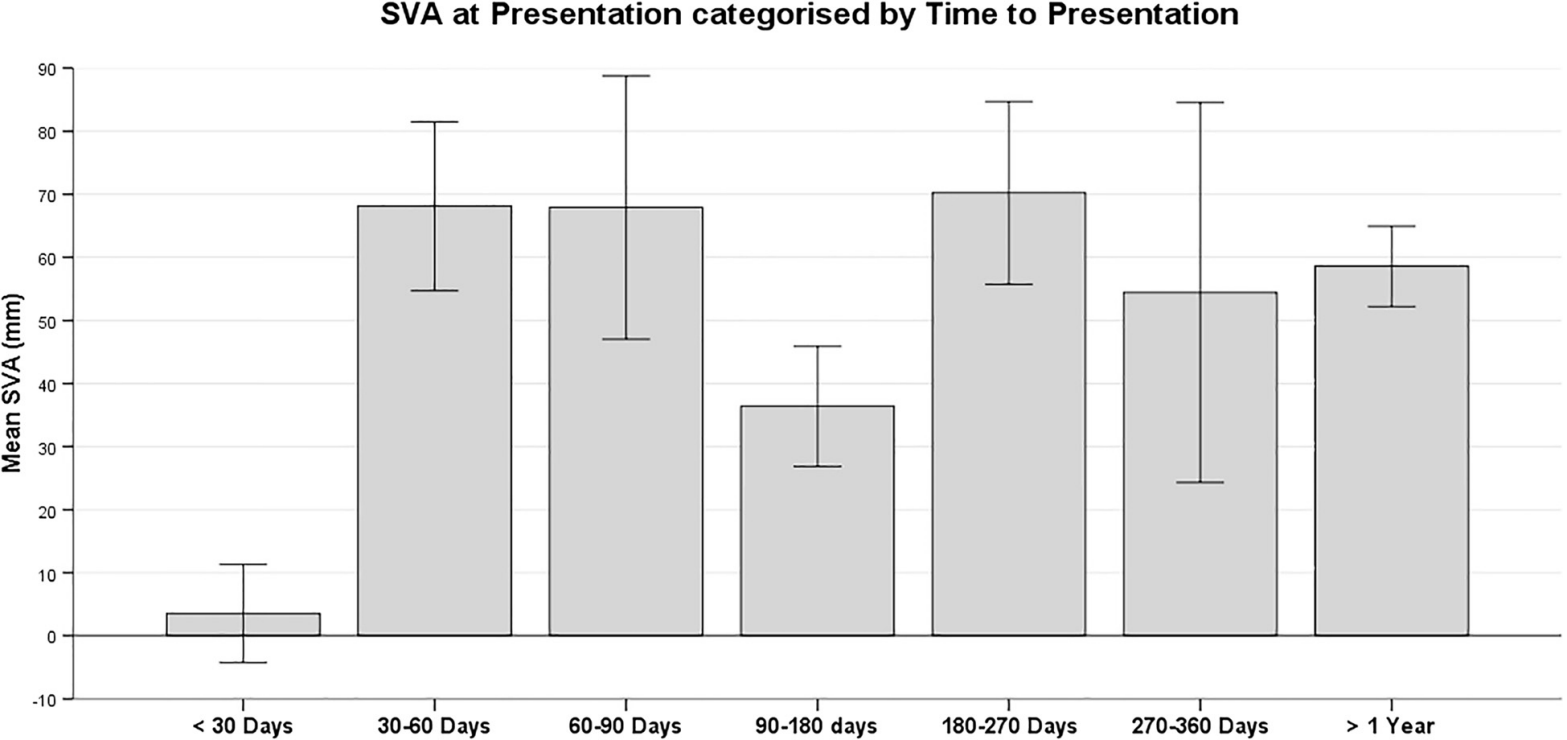
Mean Sagittal Vertical Axis by Time to Presentation. Chart demonstrating the mean sagittal vertical axis (SVA) for patients presenting between time periods as illustrated. The error bars shown illustrated the standard error. Patients presenting earlier than 30 days from diagnosis of MM had a significantly lower SVA (within the normal population range) than those presenting later

The sagittal vertical axis was 3.5 ± 25.8 mm in those presenting earlier than 30 days and significantly worse (58.2 ± 51.5 mm) in those presenting later than 30 days from diagnosis of MM (*p* < 0.001) (Fig. 1). However, only 15 patients (8.1 %) presented earlier than 30 days. Time from diagnosis to presentation did not correlate with any other radiological parameters, number or location of fractures, or SINS score. Standing radiographs were not available post-treatment to assess for change in radiological parameters.

### Management of patients

Eighty four patients (45.9 %) underwent BKP and 94 (51.37 %) were managed non-operatively, with a TLSO, in line with our local protocol as described above. Five patients (2.73 %) did not require any form of spinal treatment and are not included in analysis of follow-up. Twenty-eight patients (33.3 %) undergoing BKP had a SINS score indicating ‘impending instability’ (between 9 and 12) and in these patients the indication for BKP was pain. Thirty-two patients (34.0 %) treated in a TLSO only has a SINS score indicating an ‘unstable’ spine, but were medically unfit for surgery. Table 3 gives details, including radiological parameters of the patients in each group.

**Table 3:** Table showing the breakdown of demographics, radiological parameters and outcome scores for patients who underwent BKP and those treated with TLSO alone. It can be seen that there is no significant difference between demographics between groups, but that patients who underwent BKP had a significantly higher presentation VASB score than patients treated non-operatively. Post treatment patients treated with BKP had a better EQ-5D score than those treated with TLSO alone

	BKP patients = 84	TLSO patients = 94	Statistical significance (* = *p* < 0.05)
	Mean ± StDev/Median (range)	Mean ± StDev/Median (range)	
Age	65.2 ± 10.7 years	65.6 ± 12.4 years	*p* = 0.845
Gender:			*p* = 0.738
Male	53 (63.1 %)	57 (60.6 %)	
Female	31 (36.9 %)	37 (39.4 %)	
Disease status:			*p* = 0.670
Newly diagnosed	62 (73.8 %)	72 (76.6 %)	
Relapsed	22 (26.2 %)	22 (23.40 %)	
Chain Isotype:			*p* = 0.989
IgG	41 (48.8 %)	49 (52.1 %)	
IgA	12 (14.3 %)	10 (10.6 %)	
Light chain	12 (14.3 %)	17 (18.1 %)	
Other	5 (5.9 %)	4 (4.3 %)	
*Missing*	14 (16.7 %)	14 (14.9 %)	
Time from diagnosis to presentation	232 days (98–996 days)	141 (55–992 days)	*p* = 0.349
Number of patients who died	6 (7.14 %)	14 (14.9 %)	*p* = 0.103
Radiological parameters			
Number of fractures per patient	3 fractures (2–5 fractures)	3 fractures (1–5 fractures)	*p* = 0.282
Thoracic Kyphosis (TK)	56.4° ± 18.5°	53.2° ± 19.4°	*p* = 0.289
Lumbar Lordosis (LL)	47.2° ± 17.4°	48.6° ± 16.8°	*p* = 0.601
Sagittal Vertical Axis (SVA)	58.7 ± 49.0 mm	49.6 ± 55.0 mm	*p* = 0.288
Mid-thoracic Kyphosis (MTK)	36.9° ± 16.7°	38.8° ± 18.0°	*p* = 0.491
Thoracolumbar Kyphosis (TLK)	22.6° ± 16.2°	18.9° ± 14.6°	*p* = 0.140
SINS Score	13.0° ± 1.6°	12.4° ± 2.5°	*p* = 0.076
Patient reported outcome scores			
EQ-5D (presentation)	0.442 ± 0.220	0.463 ± 0.230	*p* = 0.526
ODI (presentation)	51.0 ± 16.6	46.2 ± 19.0	*p* = 0.085
VASB (presentation)	6.3 ± 2.3	5.5 ± 2.9	*p* = 0.031*
EQ-5D (6 weeks post treatment)	0.593 ± 0.192	0.480 ± 0.240	*p* = 0.008*
ODI (6 weeks post treatment)	42.6 ± 16.2	40.8 ± 22.1	*p* = 0.637
VASB (6 weeks post treatment)	2.8 ± 2.3	3.5 ± 3.1	*p* = 0.135

*P* values which are statistically significant have been highlighted with an '*'

For the 84 patients who underwent BKP, a median of 2 levels (range: 0–6 levels) were operated on. There were no immediate acute adverse reactions. For patients undergoing BKP, there was a statistically significant improvement in anterior-vertebral height restoration of 3.0 % ± 5.5 % (range: 0–38.1 %) (*p* < 0.001) and mid-vertebral height restoration of 3.4 % ± 6.0 % (range: 0–31.8 %) (*p* < 0.001) which was not affected by time to presentation or time from presentation to BKP. No patient lost further vertebral height post BKP. Vertebral height restoration data was available in all patients undergoing BKP.

### Patient-reported outcomes

At presentation, the mean EQ-5D was 0.435 ± 0.201, the mean ODI was 49.1 ± 16.7, and the mean VASB was 6.1 ± 2.5 for all patients. A greater number of mid-thoracic (T3-T10) fractures was found to correlate with a poorer EQ-5D score (*p* = 0.04). Increasing sagittal vertical axis correlated negatively with ODI (*p* = 0.027). Loss of lumbar lordosis (*p* = 0.016) and increased thoracolumbar kyphosis (*p* = 0.036) were correlated with a poorer EQ-5D score at follow-up, regardless of treatment. Increasing thoracolumbar kyphosis was also associated with increased VASB at follow-up in the BKP group (*p* = 0.04).

**Fig. 2:**
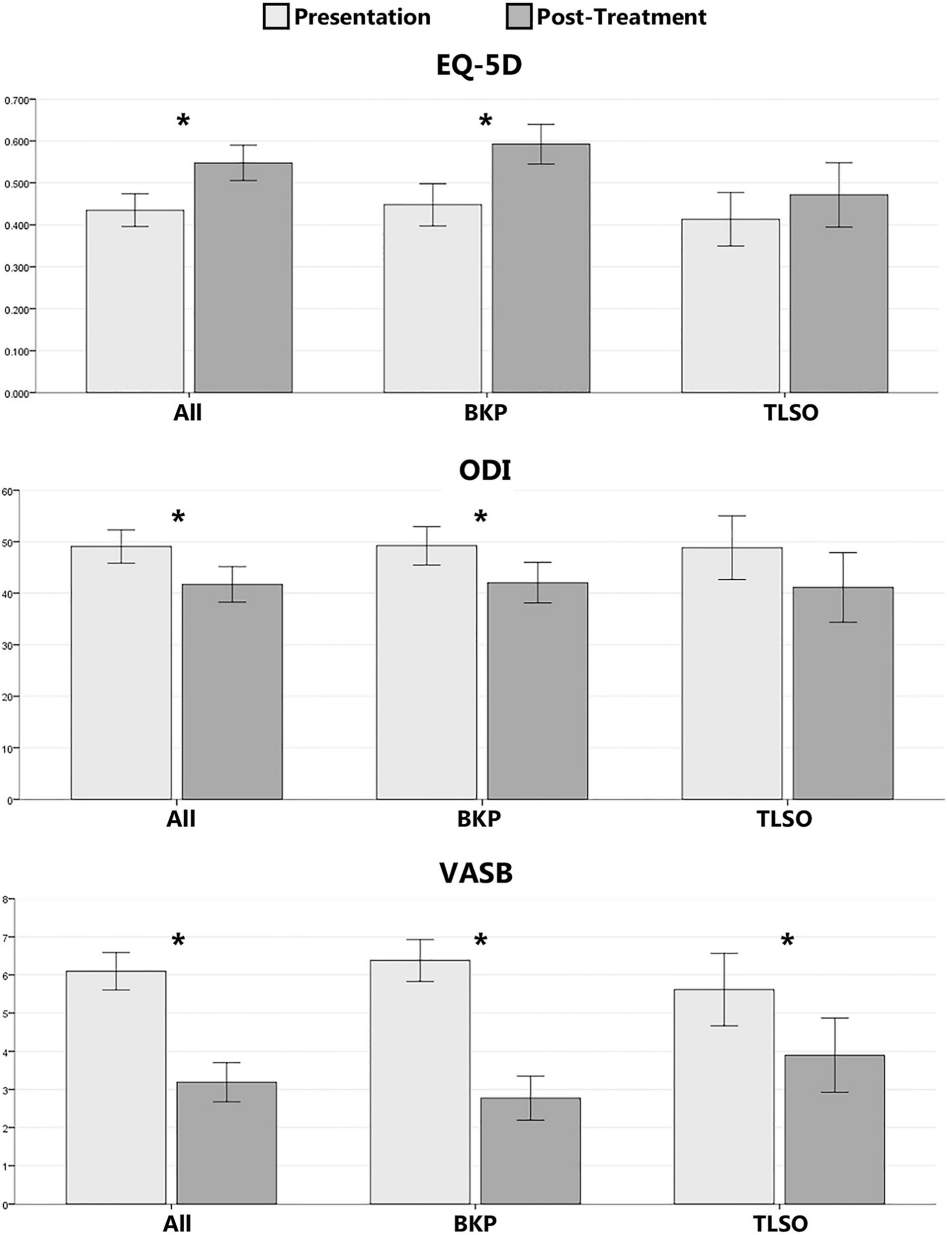
Difference in Patient Reported Scores Before and After Treatment. Results of the patient reported clinical scores recorded at presentation and follow-up 6 weeks post-intervention with 95 % confidence intervals shown. For EQ-5D a score of 1.000 represents the best possible health status and 0 represents the worst. For ODI and VASB 0, represents the best possible health state and 100 and 10 represent the worst, respectively. Annotation with ‘*’ denotes a statistically significant difference (*p* < 0.05)

The mean VASB at presentation was higher in patients undergoing BKP (6.3 ± 2.3) compared to those treated non-operatively (5.5 ± 2.9, *p* = 0.031). For all patients, at follow-up 6 weeks after treatment with a TLSO, or 6 weeks after BKP, there was a significant improvement in outcome scores. The mean post-treatment EQ-5D for all patients was 0.548 ± 0.219 (improvement of 0.107 points, *p* < 0.001), the mean ODI was 42.3 ± 18.3 (improvement of 6.8 points, *p* = 0.003), and the mean VASB was 3.2 ± 2.7 (improvement of 2.7 points, *p* < 0.001). These results are summarised in Fig. 2.

**Fig. 3:**
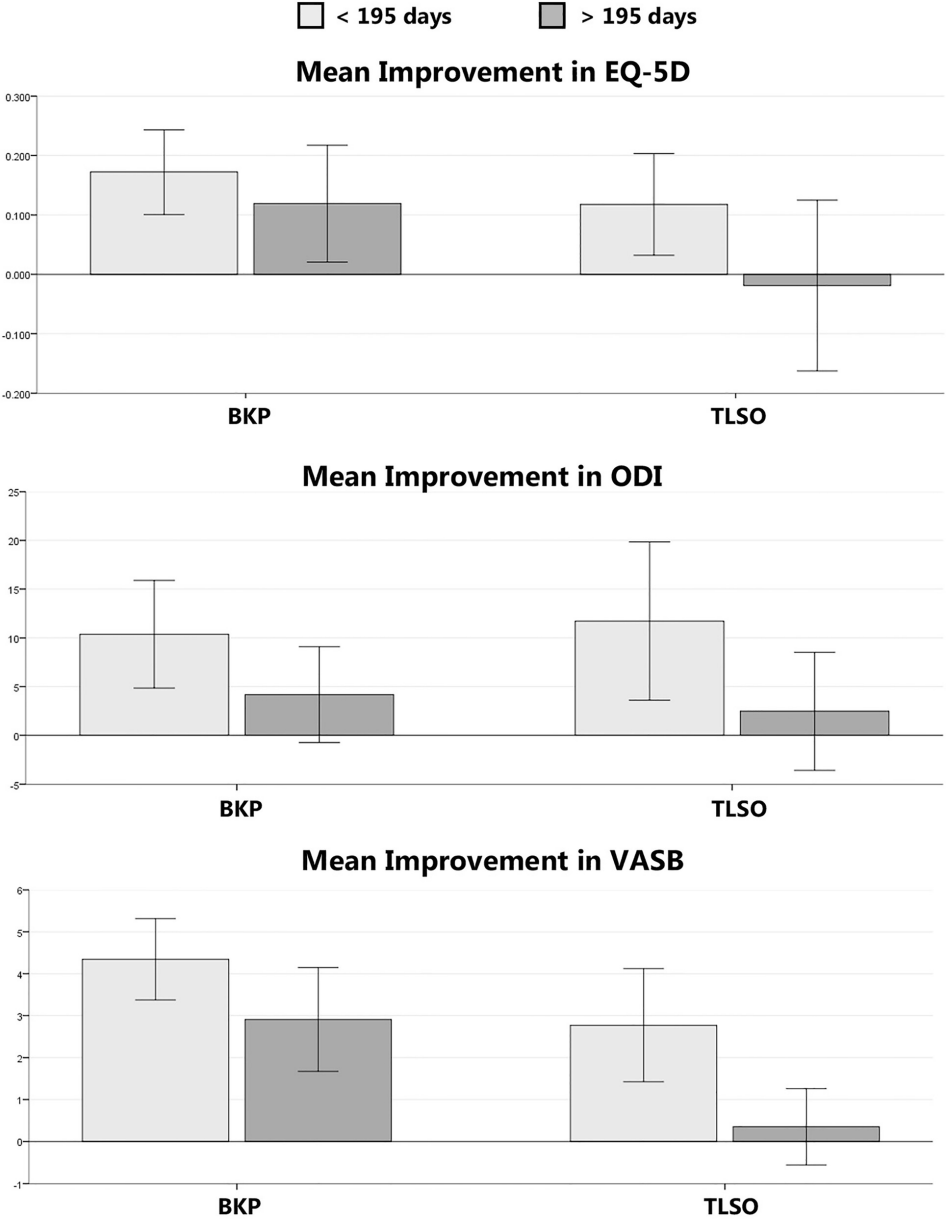
Improvement in Patient Reported Scores by Time to Presentation. This chart demonstrates the mean improvement in EQ-5D, ODI and VASB scores for Patients treated with and without BKP. Improvement was calculated as the difference between pre-treatment and post-treatment scores. The mean improvement with 95 % confidence intervals are illustrated. Patients presenting earlier than 195 days from diagnosis of MM are compared to those presenting later and were found to have a reduced benefit to treatment when presenting later, particularly in the TLSO group

In the BKP group there was a post-operative improvement in EQ-5D (*p* < 0.001, 0.144 points), ODI (*p* < 0.001, 7.2 points) and VASB (*p* < 0.001, 3.6 points) when compared to pre-operative scores. For those patients treated without BKP, there was an improvement in ODI of 7.7 points (*p* = 0.005) and in the VASB of 1.7 points (*p* < 0.001) compared to pre-treatment scores. These results are summarised in Table 3 and Fig. 3.

### Effect of disease stage and time to presentation

There was no significant difference in patient reported scores at presentation between newly diagnosed patients and those with relapsed disease. There was also no correlation between pre-existing radiological parameters and clinical benefit from either treatment group. Increasing time to presentation did not correlate with scores at presentation but on subgroup analysis it was found that patients presenting and treated within 195 days (our median time to presentation) from diagnosis had a significant improvement in clinical scores regardless of treatment.

For all patients undergoing BKP and presenting earlier than 195 days (37 patients, 44.1 %) the mean improvement in EQ-5D score was 0.171 points (*p* < 0.001), mean improvement in ODI score was 10.4 points (*p* < 0.001), and mean improvement in VASB score was 4.3 points (*p* < 0.001). In those patients presenting after 195 days (47 patients, 55.9 %) there was a reduced improvement in EQ-5D (0.122 points improved, *p* < 0.05), and VASB (3.1 points, *p* < 0.001), although still a statistically significant improvement compared to pre-treatment. However, the improvement in ODI was no longer significant (4.2 points improved, *p* = 0.09) compared to pre-treatment scores.

In those patients treated with TLSO alone, and presenting earlier than 195 days (48 patients, 51.1 %), the mean improvement in EQ-5D score was 0.118 points (*p* = 0.009), mean improvement in ODI score was 11.7 points (*p* = 0.007), and mean improvement in VASB score was 2.8 points (*p* < 0.001). However in those patients presenting after 195 days (46 patients, 48.9 %) there was no significant improvement in EQ-5D (0.016 points worse, *p* = 0.78), ODI (2.5 points improved, *p* = 0.399) or VASB (0.35 points improved, *p* = 0.422). These findings are summarised in Fig. 3 and a breakdown of clinical response by treatment group is illustrated.

## Discussion

The optimal management of spinal disease in myeloma patients continues to be a controversial area. The increasing life expectancy of these patients with the use of more effective chemotherapy regimens makes it all the more important to avoid spinal deformity early on in their management. The mechanisms by which sequential vertebral fractures and progressive spinal deformity and sagittal mal-alignment occur are examined in more detail in our supplementary data (Additional file 1).

### The effects of spinal deformity

We have assessed spinal deformity using measures of global alignment and have demonstrated that patients presenting later than 30 days from diagnosis have significantly greater deformity (as assessed by sagittal vertical axis) than those presenting earlier. Although only a small proportion of our patients presented earlier than 30 days, it is important to be aware that sagittal decompensation occurs early, and may be sudden (as illustrated in our supplementary data in Additional file 1).

The EQ-5D is a patient reported clinical outcome measure assessing pain, mobility, psychological state and ability to carry out activities of daily living. A higher score represents better function. The ODI is a clinical outcome measure where patients grade severity of symptoms and their effect on daily activities. This is assessed over 10 domains and is a well-established and validated outcome measure [26]. These clinical outcomes scores were adversely affected by deformity (sagittal vertical axis) and number of fractures at presentation. Deformity in the lumbar (lordosis) and thoracolumbar (kyphosis) regions also negatively impacted outcomes scores at post-treatment follow-up. Patients with established disease in the thoracolumbar region had a poorer response to treatment with BKP, perhaps due to ongoing mechanical instability from established deformity (illustrated in our supplementary data in Additional file 1).

We did not see a correlation between times from diagnosis to presentation and radiological or clinical parameters, apart from sagittal vertical axis. However, this may be because we had only 15 patients (8.1 %) presenting within 30 days of diagnosis. Thus we have an insufficient sample size to draw accurate conclusions about when deformity occurs and when clinical status begins to deteriorate. However, deformity is associated with adverse clinical scores at presentation and follow-up, and it is logical to aim to treat patients before deformity occurs. Treatment should be aimed at preventing sequential fractures and progressive deformity and should be commenced early. The lumbar and thoracolumbar regions are of particular importance as deformity in these regions also results in positive sagittal imbalance [27]. Treatment may be in the form of a thermoplastic TLSO or cement augmentation [18]. In addition, all patients with MM and spinal involvement should be considered for bone protection treatment, such as bisphosphonates.

### The effects of time to presentation on benefit from intervention

BKP has been reported to restore height to a fractured vertebra and improve the kyphotic deformity of the vertebral body by over 50 % if the BKP is performed within 3 months of the onset of pain in the osteoporotic population [13]. BKP and percutaneous vertebroplasty have also been used successfully in the setting of MM to prevent deformity and treat pain [15–17, 28, 29]. We found that vertebral body height was only restored by a small amount in our cohort, but importantly, no further height was lost after BKP was performed. This has also been demonstrated by previous authors [30, 31]. We cannot ascertain from our data, whether this led to a halt in overall deformity progression. Patients undergoing BKP had significantly improved EQ-5D and VASB scores regardless of time to treatment. ODI, however, only improved if patients were treated sooner than 195 days from diagnosis.

Patients treated non-operatively with a TLSO were also seen to have a statistically significant clinical improvement in EQ-5D, ODI and VASB scores if treated within 195 days of diagnosis. When presenting later, however, there was no significant benefit of treatment in a TLSO.

We offered BKP to patients with painful or unstable fractures. However, we found no difference in SINS score between our BKP and TLSO groups. This was partly because a third of patients treated in a TLSO were classed as ‘unstable’, but were not medically fit for surgery, and partly because a third of patients underwent cement augmentation for pain rather than instability. The latter is reflected in the difference observed between VASB at presentation in BKP and non-BKP groups.

Our results indicate that regardless of treatment, early intervention benefits patients. Delay in treatment lessens this benefit, particularly in patients managed without BKP. It is unclear from our data whether operative or non-operative treatment is superior when instituted early, however, BKP may be more suitable for treatment of patients with delayed presentations. This may, in particular, adversely impact those patients with delayed presentations who are not medically fit for surgery.

### Strengths and weaknesses

This was a single centre series with a large cohort of patients managed according to the same clinical protocols. Thus we are able to perform multiple correlations. However, as this was a retrospective analysis of data, it is difficult to determine the exact duration of symptoms and the period over which multiple VCFs occurred. It is also difficult to assess the progression of the spinal deformity with sequential VCFs. The time from onset of back pain to presentation could not be reliably assessed for several reasons. Patients may have had back pain for many months prior to diagnosis, or have developed back pain related to interval fractures or marrow infiltration, particularly in those with relapsed MM. Time from onset of back pain to presentation may indeed be an important parameter which could be addressed in future studies examining the relationship between time from onset of back pain to development of deformity and adverse clinical outcome in patients with MM.

Patients were treated with BKP and a TLSO, or a TLSO alone depending on the degree of pain and stability. In general patients with higher VASB scores and less stable vertebral fractures at presentation were offered BKP. This makes direct comparisons of outcomes between these groups of patients problematic as they may be inherently different. Nevertheless, some patients with pain/less stable vertebral fractures were treated with a TLSO only and significant improvement was seen in post-treatment VASB in both groups.

This was a retrospective study including all newly referred myeloma patients with adequate data and we did not apply specific exclusion criteria. Although we examined the relationship between demographics, time to presentation and outcome, other factors may account for differences between groups.

Our follow-up is reported at 6 weeks post-treatment as we do not have sufficient data from 3 and 6 month follow-up time points. Patients often did not attend subsequent appointments for varying reasons, and some developed additional lesions which could confound the outcomes [15, 29]. It is our experience that patients cannot reliably distinguish between symptoms from treated and new fractures. For this reason we do not routinely record scores after 6 weeks and less than 10 % of included patients had scores recorded at 3 and 6 months. We have therefore not reported longer term follow-up data. However, in the absence of further VCFs, the improvement in pain and outcome scores following cement augmentation has been shown to persist up to 5 years [16].

Existing literature on spinal myeloma focuses on cement augmentation of individual VCFs but does not describe overall spinal deformity (sagittal mal-alignment) nor the impact of time to presentation on efficacy of treatment. Existing literature also does not report on the results of non-operative treatment of spinal myeloma with a TLSO.

## Conclusions

Our results demonstrate that patients with spinal involvement of MM develop significant deformity. Health related quality of life scores are poor in the setting of these deformities, particularly when affecting the thoracolumbar junction. Treatment at a specialist spinal myeloma unit improves clinical scores but those patients with delayed presentation, or significant deformity may benefit less from treatment. This reduced benefit is particularly seen in patients treated non-operatively. We suggest that all patients with MM and back pain or an early clinical spinal deformity be screened for spinal lesions as a matter of urgency and urgent referral to a specialist spinal myeloma unit be considered.

## Abbreviations

BKP, balloon kyphoplasty; EQ-5D, Euro-Qol 5 Dimensions; MM, multiple myeloma; ODI, oswestry disability index; SINS, spinal instability neoplastic score; SVA, sagittal vertical axis; TLSO, thoraco-lumbar-sacral orthosis; VASB, visual analogue score for the back; VCF, vertebral compression fracture

## Additional file

Additional file 1: Description of Data: Mechanism of vertebral compression fractures and sagittal spinal alignment. Mechanism of progressive spinal deformity. Measurements of radiographic parameters. (DOCX 1192 kb)
