# Supplementary material for: Spinal disease in myeloma: cohort analysis at a specialist spinal surgery centre indicates benefit of early surgical augmentation or bracing
Source: BMC Cancer. 2016 Jul 11;16:444. doi: 10.1186/s12885-016-2495-7 (PMC4939590; doi:10.1186/s12885-016-2495-7)
Supplement: Additional file 1: — Description of Data: Mechanism of vertebral compression fractures and sagittal spinal alignment. Mechanism of progressive spinal deformity. Measurements of radiographic parameters. (DOCX 1192 kb) [file 12885_2016_2495_MOESM1_ESM.docx]

**Supplementary Data:**

***Mechanism of Vertebral Compression Fractures and Sagittal Spinal Alignment***

In the osteoporotic literature presence of one VCF has been shown to be a risk factor for development of further VCFs, in turn leading to an increased thoracic kyphosis.[1-3] Kyphosis occurs as VCFs are typically wedge shaped with a proportionally greater loss of anterior vertebral body height (Supplementary Figure 1). This increases the strain placed on the anterior aspect of adjacent vertebral bodies which may then fail sequentially, further exacerbating the deformity. [4]

The global alignment of the spinal column is assessed in both the coronal and sagittal planes. The lumbar spine is normally lordotic and the thoracic spine is kyphotic. In a balanced spine the C7 vertebra, which can be taken to represent the centre of gravity of the spine, sits directly in line with the sacrum. When analysing a standardised lateral standing radiograph of the spine, the sagittal alignment is therefore determined by drawing a vertical plumb line down from C7. If this line passes within 40mm of the posterior superior aspect of S1 the spine may be considered to have normal overall sagittal alignment.[5] This measurement is termed sagittal vertical axis and is demonstrated in Supplementary Figure 2.

***Mechanism of progressive spinal deformity***

To compensate for the kyphosis produced by VCFs and maintain normal sagittal alignment a patient will arch their back, increasing the lumbar lordosis. This enables the patient to maintain a horizontal gaze but requires increased work of the paraspinal muscles to maintain sagittal alignment. [6, 7] The increased lordosis may overload the facet joints in the lumbar spine resulting in degenerative changes and pain. [8] This may not be obvious from plain radiographs or MR scans but single positron emission computed tomography (SPECT) scans may be used in conjunction with computed tomography (CT) to confirm the diagnosis. [9]

If VCFs are multiple, or kyphosis more pronounced, eventually the spinal extensor muscles will fatigue and the patient will not be able to maintain the lordosis required to achieve normal sagittal alignment. Instead the patients will adopt an increasingly kyphotic posture. This sequence of events is demonstrated in Figure 3. Sagittal vertical axis is a surrogate marker of this phenomenon and when it increases beyond the norm, it is termed positive sagittal malalignment and is associated with significant pain and disability. [10]

It is important to note that although increased thoracic kyphosis is associated with pain, a hump-back deformity, and may cause respiratory compromise, it may not cause an increase in sagittal vertical axis as patients may compensate. Fractures of the thoracolumbar spine however cause a proportionately greater shift forward in the centre of gravity of the spine. [11] This is illustrated in Supplementary Figure 2.

***Measurements of Radiographic Parameters***

Supplementary Figure 4 demonstrates some of the radiographic measurements we conducted to assess spinal deformity. Supplementary Figure 5 shows the method of calculating degree of collapse of vertebral compression fractures.

***References***

1. Baek SW, Kim C, Chang H. The relationship between the spinopelvic balance and the incidence of adjacent vertebral fractures following percutaneous vertebroplasty. Osteoporos Int. 2015;26(5):1507-13.

2. Maejima H, Takeishi K, Sunahori H, Yamawaki A, Nakajima K, Yoshimura O. The Relationship between Postural Deformation and Standing Balance in Elderly Person. J Jpn Phys Ther Assoc. 2004;7(1):7-14.

3. Yokoyama K, Kawanishi M, Yamada M, Tanaka H, Ito Y, Kawabata S, et al. Postoperative change in sagittal balance after Kyphoplasty for the treatment of osteoporotic vertebral compression fracture. Eur Spine J. 2015;24(4):744-9.

4. Papanastassiou ID, Phillips FM, Van Meirhaeghe J, Berenson JR, Andersson GB, Chung G, et al. Comparing effects of kyphoplasty, vertebroplasty, and non-surgical management in a systematic review of randomized and non-randomized controlled studies. Eur Spine J. 2012;21(9):1826-43.

5. Jackson RP, Kanemura T, Kawakami N, Hales C. Lumbopelvic lordosis and pelvic balance on repeated standing lateral radiographs of adult volunteers and untreated patients with constant low back pain. Spine (Phila Pa 1976). 2000;25(5):575-86.

6. Yuan HA, Brown CW, Phillips FM. Osteoporotic spinal deformity: a biomechanical rationale for the clinical consequences and treatment of vertebral body compression fractures. J Spinal Disord Tech. 2004;17(3):236-42.

7. Cho Y, Lee G, Aguinaldo J, Lee KJ, Kim K. Correlates of bone mineral density and sagittal spinal balance in the aged. Ann Rehabil Med. 2015;39(1):100-7.

8. Jentzsch T, Geiger J, Konig MA, Werner CM. Hyperlordosis Is Associated With Facet Joint Pathology At The Lower Lumbar Spine. J Spinal Disord Tech. 2013.

9. Kwok IH, Butler JS, Selvadurai S, Molloy S. The utility of single photon emission computed tomography/computed tomography (SPECT/CT) fusion imaging in the diagnosis of a vertebral compression fracture in multiple myeloma. Spine J. 2015.

10. Glassman SD, Bridwell K, Dimar JR, Horton W, Berven S, Schwab F. The impact of positive sagittal balance in adult spinal deformity. Spine (Phila Pa 1976). 2005;30(18):2024-9.

11. Glassman SD, Berven S, Bridwell K, Horton W, Dimar JR. Correlation of radiographic parameters and clinical symptoms in adult scoliosis. Spine (Phila Pa 1976). 2005;30(6):682-8.

**Supplementary Figure 1:**

This figure illustrates the compressive force acting on the spinal column, resulting in a series of vertebral compression fractures (VCFs). There is a proportionately greater loss of anterior vertebral height, resulting in localised kyphosis. This causes increases strain on adjacent vertebral bodies which may also develop VCFs. Kyphosis progresses as number of VCFs increases.


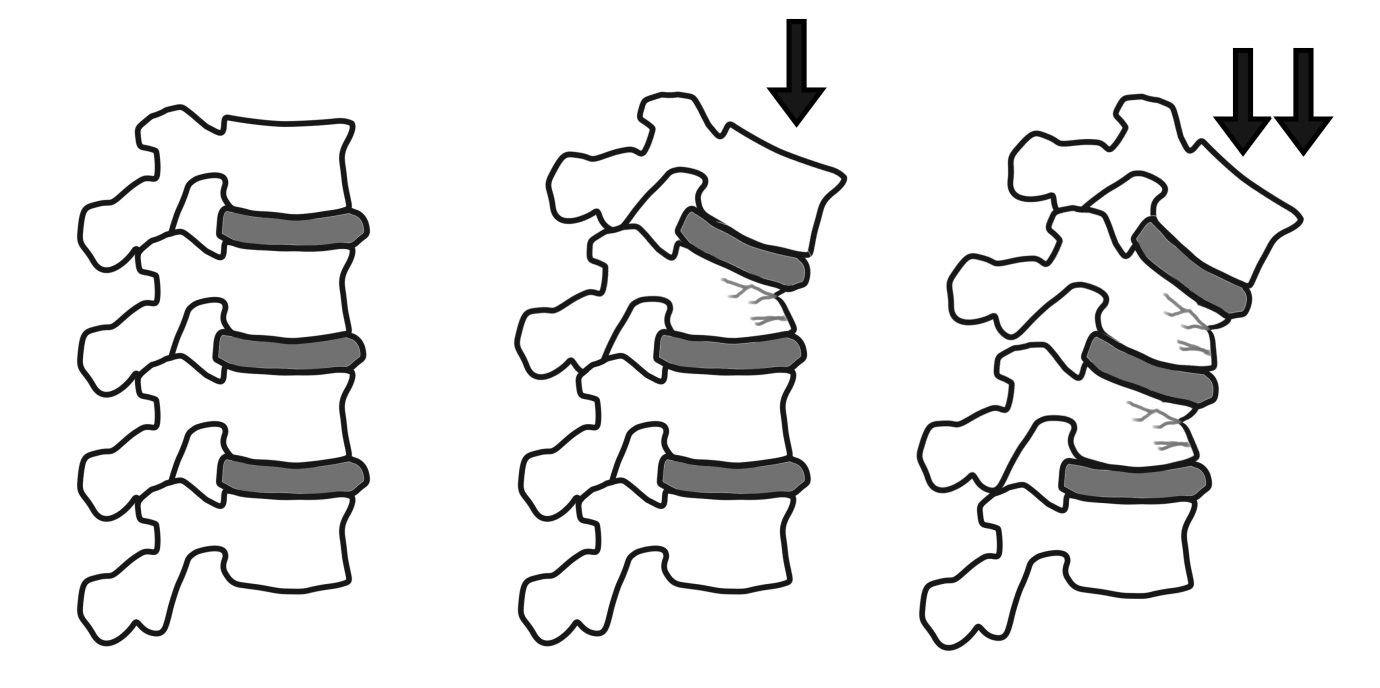


**Supplementary Figure 2:**

Clinical photographs and lateral standing radiographs of the spine demonstrating the C7 plumb line (drawn in white) and the sagittal vertical axis (drawn in black). Figure 2A shows a patient with mid thoracic kyphosis. Although this may impact on respiratory function, the sagittal vertical axis is not increased as the patient has managed to compensate by increasing their lumbar lordosis (Radiograph in Figure 2B). The patient has developed a pressure sore on the most prominent point of his kyphosis. Figure 2C shows a patient who has thoracolumbar kyphosis and who is compensating for this by flexing his knees. When the patient straightens his knees his centre of gravity shifts forward (Figure 2D). The corresponding radiograph of Figure 2D is shown in Figure 2E and demonstrates the increased sagittal vertical axis.

**
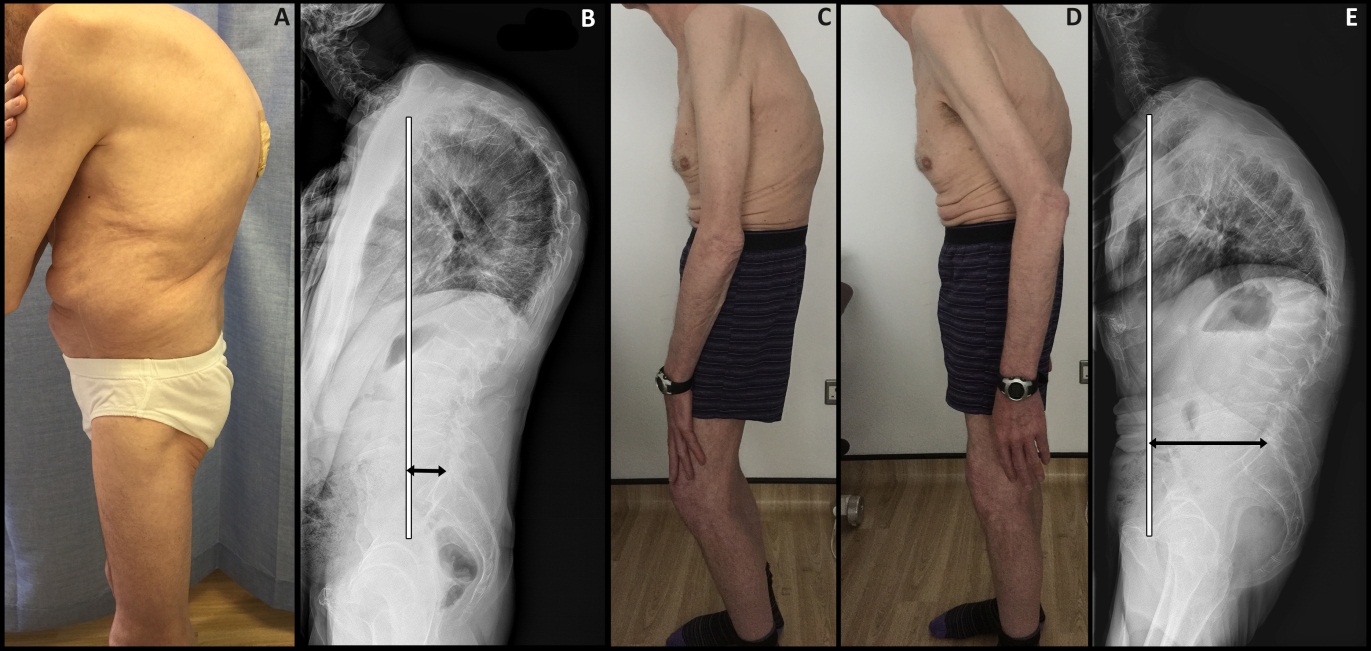
**

**Supplementary Figure 3:**

Figure 3A demonstrates an MR scan of a patient with a fracture of the T9 vertebra causing localised kyphosis. Figure 3B shows the SPECT/CT imaging demonstrating overloaded facet joints of L2-3. The gold region represents increased isotope uptake which is therefore an area of increased mechanical stress and potentially a pain generator. This occurs due to hyperlordosis in an attempt to correct for kyphosis. Eventually pain and fatigue make it difficult to maintain an upright posture and kyphosis develops, which may be severe (Figure 3C). Repeat MR scan in Figure 3D demonstrates that multiple adjacent vertebral bodies have developed fractures and kyphosis as well. This process can occur in a matter of weeks to months.


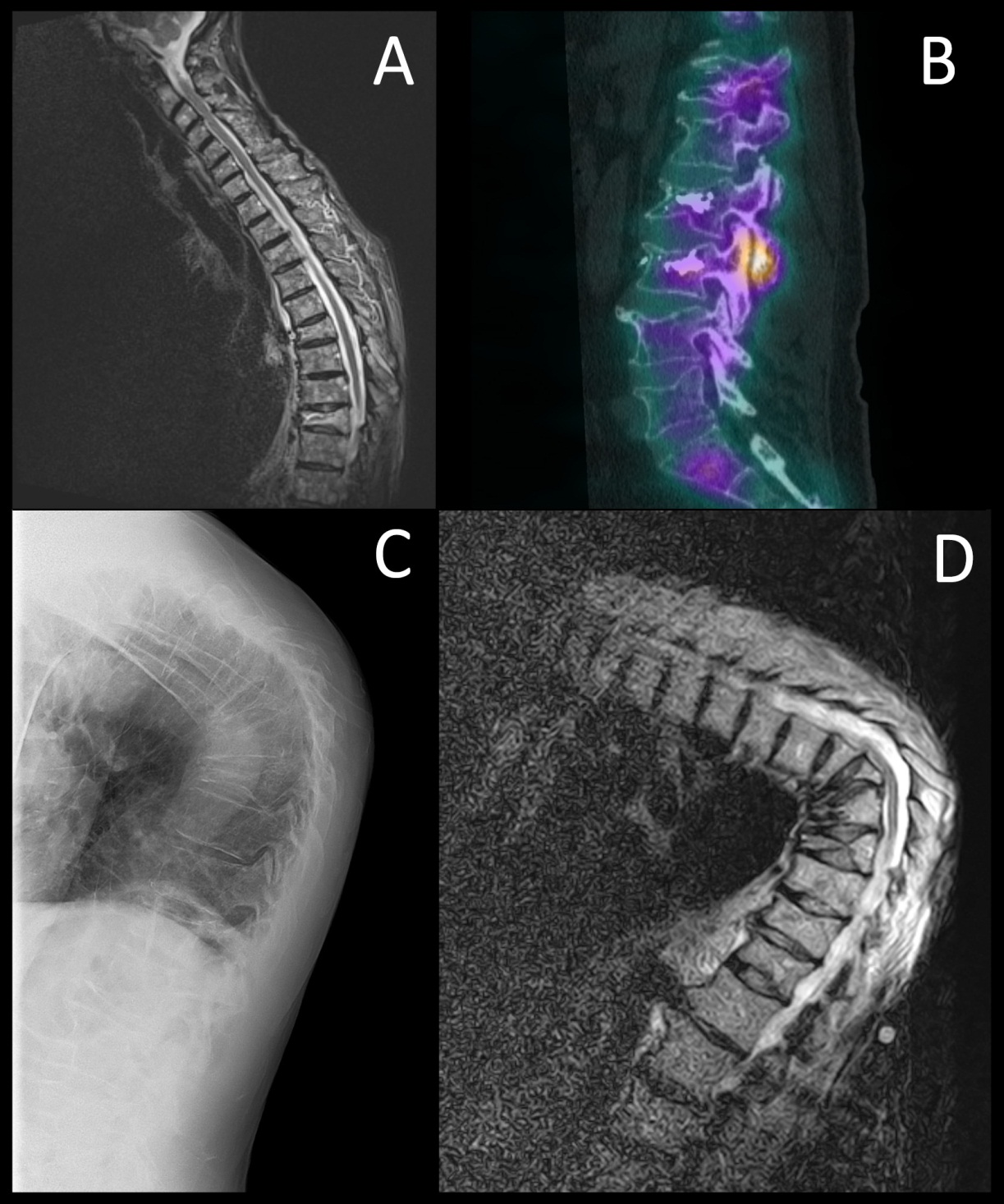


**Supplementary Figure 4:**

Standardised, lateral, erect radiograph of the spine. Thoracolumbar kyphosis (TLK) is shown, measured between the T10 to the L2 vertebral bodies. This should normally be less than 10°, but in this case is increased due to fractures of T10, T11 and L1. The centre of gravity of the spine has therefore shifted forward and the sagittal vertical axis (SVA), depicted with dotted lines, has increased (normally less than 40 mm).

**
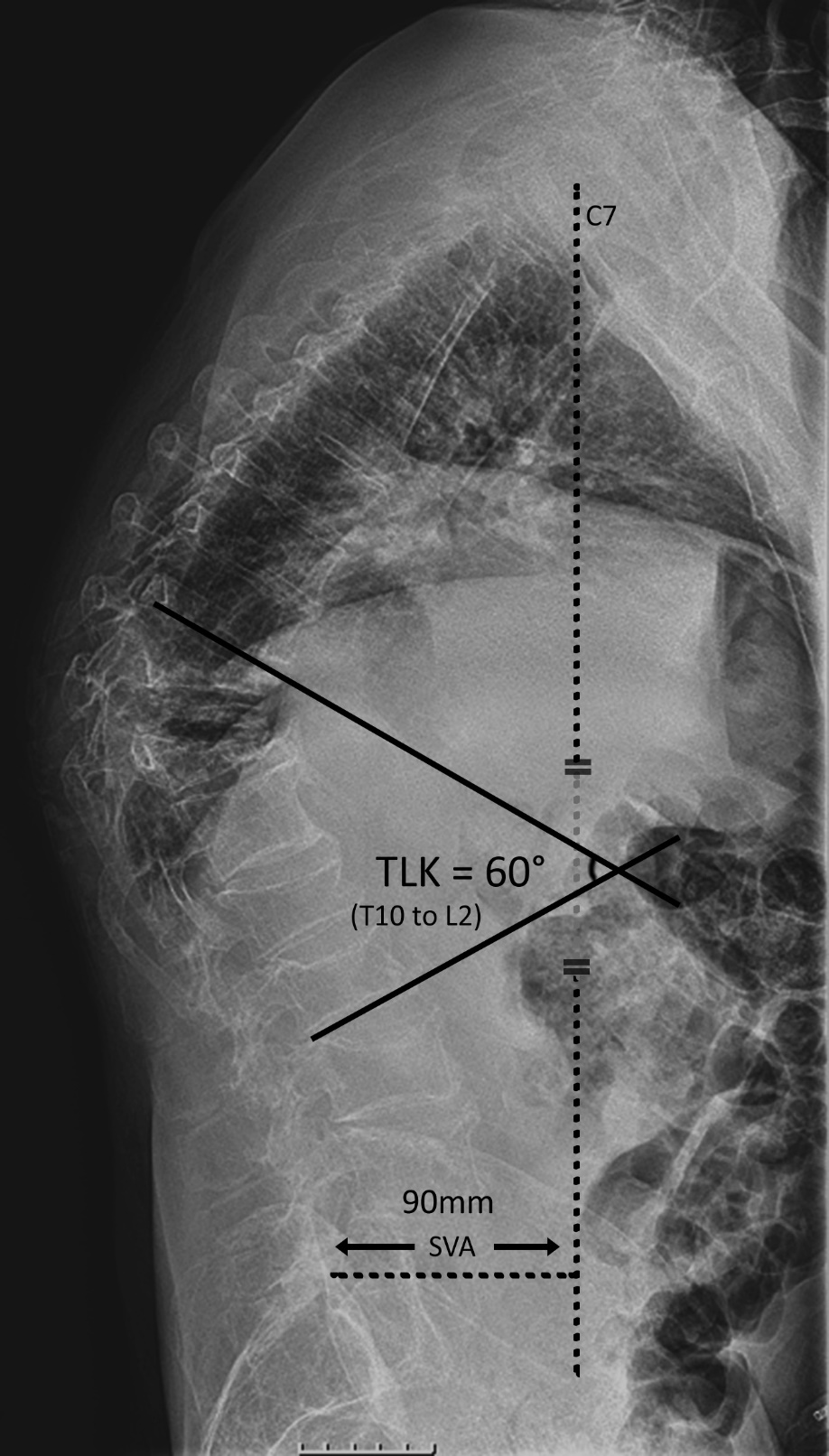
**

**Supplementary Figure 5:**


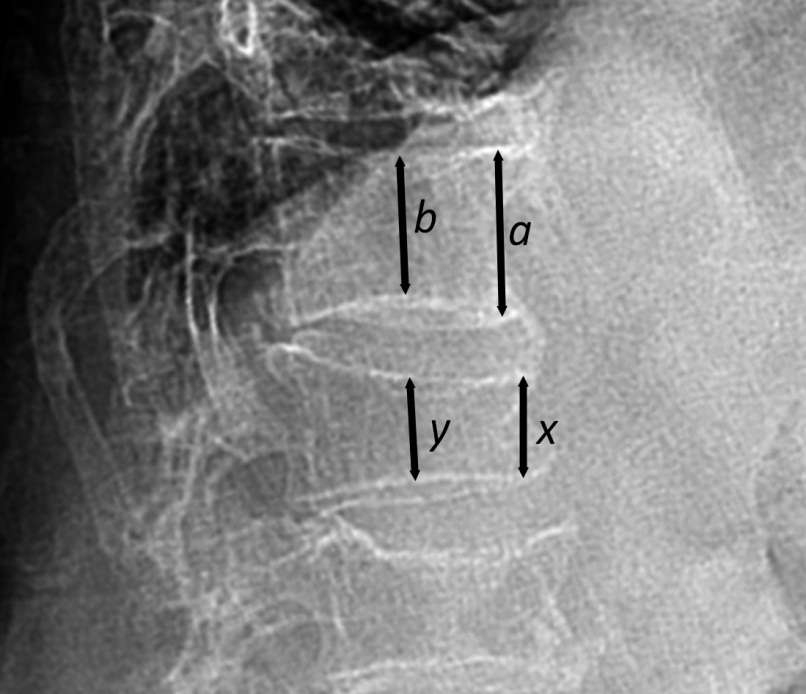
This figure illustrates the measurements taken when assessing vertebral height. Depicted is a radiograph taken pre-kyphoplasty. Distances ‘a’ and ‘b’ represent the anterior and middle vertebral body heights respectively, measured on an adjacent unaffected vertebra. Heights ‘x’ and ‘y’ represented the anterior and middle vertebral body heights respectively, measured on the operated vertebra. These measurements were repeated on post-operative radiographs.
